# Counting to infinity, successive addition, and the length of the past

**DOI:** 10.1007/s11153-022-09843-0

**Published:** 2022-08-27

**Authors:** Mohammad Saleh Zarepour

**Affiliations:** Department of Philosophy, School of Social Sciences, The University of Manchester, Humanities Bridgefoot Street Building, Oxford Road, Manchester, M13 9PL UK

**Keywords:** Kalām Cosmological Argument, Beginning of the universe, The Successive Addition Argument, Dretske’s objection, Counting to infinity, Finitism

## Abstract

The Successive Addition Argument (SAA) is one of the arguments proposed by the defenders of the Kalām Cosmological Argument to support the claim that the universe has a beginning. The main premise of SAA states that a collection formed by successive addition cannot be an actual infinite. This premise is challenged by an argument originally proposed by Fred Dretske. According to Dretske’s Argument (DA), the scenario of a counter who starts counting numbers and never stops can provide a counterexample to the main premise of SAA. I argue that neither DA nor its past-oriented counterpart—which discusses the scenario of a counter who has always been counting negative integers from the infinite past—can play a decisive role in our evaluation of the strength of the arguments that are intended to establish the finitude of the past based on the impossibility of an actually infinite number of successive additions.

The Kalām Cosmological Argument (KCA) aims to establish that the universe has a cause based on the following premises: (a) the universe has a beginning; and (b) everything that has a beginning has a cause.^1^ To justify (a), the proponents of KCA appeal to various arguments, one of which is the Successive Addition Argument (SAA), which runs as follows:

***The Successive Addition Argument (SAA)***

(S1) The temporal series of events is a collection formed by successive addition.

(S2) A collection formed by successive addition cannot be an actual infinite.

Therefore:

(S3) The temporal series of events cannot be an actual infinite.^2^

If the universe has no beginning, then the temporal series of past events is actually infinite. But if SAA is sound, then the temporal series of past events, which is a subseries of the temporal series of all events, cannot be actually infinite. This indicates that the universe cannot be beginningless. Thus, if SAA is sound, (a) would be true. To question the soundness of SAA, some opponents of KCA have argued against the truth of (S2) by appealing to an argument originally put forward by Fred Dretske.^3^ He argues that if someone, call him George, starts counting numbers and never stops, he will actually count to infinity. So, he can form an actually infinite collection (i.e., the collection of George’s counting events) by successive addition. This contradicts (S2). Thus, if Dretske’s Argument (DA) is successful, SAA would fail. To see how this line of argument is supposed to work, consider the following reconstruction of DA by Alex Malpass:

***Dretske’s Argument (DA)***

(D1) It is possible that George starts counting now and will never stop.

(D2) If George starts counting now and will never stop, then for each natural number, n, George will count n.

(D3) If George will count each natural number, then George will count ℵ_0_-many numbers.

Therefore:

(D4) It is possible that George will count ℵ_0_-many numbers.^4^

Now, if we consider Craig’s definition of an actual infinite, we see that counting ℵ_0_-many numbers is indeed tantamount to forming an actual infinite by successive addition:

An actual infinite is a collection of definite and discrete members whose number is greater than any natural number 0, 1, 2, 3... This sort of infinity is used in set theory to designate sets that have an infinite number of members, such as {0, 1, 2, 3...}. The symbol for this kind of infinity is the Hebrew letter aleph: ℵ. The number of members in the set of natural numbers is ℵ_0_. By contrast, a potential infinite is a collection that is increasing toward infinity as a limit but never gets there. The symbol for this kind of infinity is the lemniscate: ∞.^5^

If George will count ℵ_0_-many numbers, he will create a collection of counting events whose members can be put in one-to-one correspondence with the set of natural numbers. Thus, George’s counting activity will form an actually infinite collection by successive addition.^6^ This apparently contradicts the second premise of SAA—i.e., (S2). As a result, if DA is sound, SAA would be unsound. In this short paper, I argue that, despite its *prima facie* plausibility, this argumentative manoeuvre against SAA is illegitimate and has no chance of success.

Note that SAA talks about series of events that happen in consecutive temporal intervals of the same length.^7^ Accordingly, the counting events about which DA talks must similarly happen in consecutive temporal intervals of a certain length. Otherwise, DA (even if it is sound) cannot provide a counterexample to (S2). That is why the proponents of DA usually make it explicit that counting events are supposed to occur at a constant rate (e.g., one counting event per second).^8^ But this means that the first premise of DA cannot be true unless it is already accepted that the future can be endless. It is possible that George now starts counting at a constant rate and will never stop only if it is possible that the future has no end. One who finds the possible infinitude of the future dubious would never assent to the truth of (D1). Thus, it seems obvious that DA takes it for granted that the future is possibly infinite. Accordingly, DA seems to presuppose that the temporal series of future events, in particular, and the temporal series of all events, in general, can in principle be actual infinites. This indicates that the possible infinitude of the temporal series of events is a hidden premise of DA. Thus, it seems to be illegitimate to employ DA to argue against an argument like SAA, whose conclusion is the impossible infinitude of the temporal series of events.

In general, an argument that presupposes P is not eligible to be employed as an objection to an argument for the negation of P (i.e., ~ P). To presuppose P to show the unsoundness of an argument for ~ P is simply to beg the question of the unsoundness of that argument. For this reason, DA, which presupposes the possible infinitude of the temporal series of future events, has no force either against SAA or against any argument for the impossible infinitude of the temporal series of future events. When the question of whether or not the temporal series of events can be infinite is not yet settled, we cannot criticize an argument for a specific position (regarding that question) by appealing to an argument that simply presupposes the negation of that position. Such an attack is indeed a nonstarter. For this reason, we cannot legitimately appeal to DA to attack SAA.

To block the above objection, those who believe that DA undermines SAA might suggest that SAA must be read as an argument exclusively about the temporal series of past events, rather than the temporal series of events in general. This is because SAA is intended to establish the finitude of the past, which is the first premise of KCA. On this suggestion, SAA must be taken as a slightly abbreviated version of the following argument (which is obtained from adding the *italic* terms to SAA):

SAA*

(S*1) The temporal series of *past* events is a collection formed by successive addition.

(S*2) A collection formed by successive addition cannot be an actual infinite.

Therefore:

(S*3) The temporal series of *past* events cannot be an actual infinite.

The proponents of this idea could argue that DA can legitimately be employed to refute SAA*, even if its employment against SAA is illegitimate. This is because DA does not presuppose the possible infinitude of the temporal series of past events. In other words, DA does not presuppose the negation of (S*3). As a result, the employment of DA against SAA* is not problematic in the same sense that its employment against SAA is. On the one hand, DA shows that the collection of George’s counting events is an actually infinite collection formed by successive addition. So, it provides a counterexample to (S*2), which is identical to (S2). On the other hand, it does not presuppose the negation of (S*3). Therefore, it seems that DA can legitimately refute SAA*. Even if DA cannot be used against an argument for the impossible infinitude of the temporal series of all or future events, its use against an argument for the impossible infinitude of the temporal series of past events seems to be unproblematic. Moreover, what is in question in KCA is the infinitude of the past, not the infinitude of the future or the infinitude of time in general. So, what the advocates of KCA really mean by SAA must be something similar to SAA*. But DA can legitimately be proposed against SAA*.

Regardless of whether an opponent of an argument is allowed to interpret it on behalf of its proponent, the above strategy fails because the friends of KCA can provide an analysis of the notion of *being-formed-by-successive-addition* based on which the conclusion of DA is perfectly compatible with (S*2). To see how this is possible, we need to engage with *the accumulation objection* to DA, which is recently discussed by Alex Malpass.^9^

Imagine the scenario of dropping marbles into a jar one by one. It seems obvious that even if it is possible to drop an actually infinite number of marbles into the jar one by one, the number of marbles accumulated in the jar is always finite. Now, one might think that, for the same reason, the number of the events accumulated in the jar of the past is always finite. This is true regardless of whether or not the number of future events is actually infinite, or so a defender of SAA* might claim. Malpass suggests that this objection might be motivated by the following analysis of the notion of *being-formed-by-successive-addition*:

***Analysis***

A collection is ‘formed by successive addition’ if and only if.

*Succession*: The addition of each element to the collection succeeds the previous one (apart from the first, if there is one);

*Accumulation*: There is a totality that accumulates from the succession.^10^

Malpass seems to believe that the condition of *Accumulation* is satisfied only when all the elements of the accumulated totality exist together. He argues that “the temporal series is disanalogous to dropping marbles into a jar” because on the presentist view of time that Craig endorses, “there is no point at which consecutive past events, such as George’s counting events, ever exist together.”^11^ Malpass in the end concludes that:

[E]ither the notion of successive addition includes *Accumulation*, in which case the numbers George has counted do not qualify, or it does not include *Accumulation*, in which case the numbers that George will count do qualify. What one does not seem to be able to do, however, is have one notion which applies to the numbers he has counted but not to the numbers he will count.^12^

I agree with Malpass that the past events are not accumulated in the sense that the marbles in the jar are. If presentism is true, then the past events do not exist all together. In this sense, their status differs from that of the marbles in the jar. Thus, if a collection satisfies the condition of *Accumulation* only when its elements exist all together, then the temporal series of past events does not satisfy this condition. Indeed, it seems that the conjunction of *Analysis* and presentism casts doubt upon not only (S*2) but also (S*1). According to (S*1), the temporal series of past events is a collection formed by successive addition. Now, if the satisfaction of *Accumulation* is a necessary condition for a collection to be formed by successive addition, then all the past events must exist together. However, this obviously contradicts presentism (and any other view of time on which it is not the case that all the moments of the past time exist together). Thus, a presentist like Craig cannot consistently defend the soundness of SAA* by endorsing *Analysis*.

I also agree that if the notion of successive addition includes only *Succession* (without *Accumulation*), then the temporal series of future events can be taken as a collection formed by successive addition. This is because this collection satisfies the condition of *Succession*, even though it is not accumulated in the above sense. Nevertheless, I think we have a notion which applies to past but not future events. Like the formation process of the series of past events, the formation process of the series of future events is constituted by successive addition. But by contrast with the former series, whose formation process is *completed*, the formation process of the latter series will never be *completed*. This difference can best be captured by the following analysis:

Analysis*

The formation process of a collection is completed by successive addition if and only if.

*Succession*: The addition of each element to the collection succeeds the previous one (apart from the first, if there is one);

*Completion*: There is a moment of time *t* such that every element of the collection exists at *t* or some moment before *t*.

If we endorse Analysis*, then the temporal series of past events would be a collection whose formation process is completed by successive addition. But the formation process of the collection of George’s future counting events will never be completed by successive addition. This is because although this collection satisfies *Succession*, it does not satisfy *Completion*. All the past events have happened before the present time. But there is no moment of time up until which all George’s future counting events will have happened. Based on these observations, the defenders of KCA can argue that SAA* must be understood as a paraphrase of the following argument for the beginning of the past:

SAA**

(S**1) The temporal series of past events is a collection whose formation process is completed by successive addition.

(S**2) A collection whose formation process is completed by successive addition cannot be an actual infinite.

Therefore:

(S**3) The temporal series of past events cannot be an actual infinite.

The conclusion of this argument is identical to that of SAA*. If SAA** is sound, then the universe has a beginning. This is because, if the universe is beginningless, then the temporal series of past events is an actual infinite, in the sense that these events can be put in one-to-one correspondence with natural numbers.^13^ But the conclusion of SAA** rejects the possibility of the existence of such an actually infinite series. Thus, if SAA* is sound, the universe must have a beginning. More importantly for our purposes, if we understand the premises of SAA** in terms of Analysis*, then DA cannot question the soundness of SAA**. In particular, DA does not provide a counterexample to (S**2). Even the most ardent proponents of DA admit that although George will count every number, he will never have counted every number.^14^ This means that the formation process of the collection of George’s counting events will never be completed. So, this collection cannot provide a counterexample to (S**2). And this fact does not hinge on whether or not we uphold presentism. It is compatible with all the well-known theories of time (e.g., presentist, externalist, and growing block theories of time) that the series of George’s future counting events does not satisfy *Completion*.

It is worth emphasizing, once again, that I do not reject that the collection of George’s future counting events can be actually infinite. Since these events can be put in one-to-one correspondence with the set of natural numbers, their collection satisfies Craig’s definition of an actual infinite. Nevertheless, the formation process of this collection does not satisfy *Completion*. Consequently, this collection cannot be taken as a collection whose formation process is completed by successive addition. Thus, the actual infinitude of this collection is compatible with the truth of (S**2). Furthermore, the point I have tried to make is entirely different from the simple-to-perfect objection that is discussed in the literature. According to that objection, one might deny that George will count all the natural numbers by arguing that if it is the case that he will count all the natural numbers, it will also be the case that he will have counted all the natural numbers. But since the latter claim is false, the former must be false as well.^15^ In contrast to such an objector, I agree with Malpass that, on the one hand, George will count all the natural numbers and, on the other hand, this fact does not need to entail that he will have counted all the natural numbers.

SAA** seems to be an argument for the finitude of the past from the impossibility of an actually infinite number of successive additions whose soundness cannot be challenged by DA. If one reacts to the fact that DA cannot be legitimately used against SAA by suggesting that SAA must be understood as SAA*, then the defenders of KCA can interpret SAA* as SAA** and argue, based on Analysis*, that the conclusion of DA is perfectly compatible with the truth of (S**2) and the soundness of SAA** in general.

Now an opponent of KCA might insist that we can easily produce a counterexample to (S**2) even if it is understood based on Analysis*. For example, one might suggest that by slightly changing Dretske’s example and considering George as a counter who has always been counting negative integers from the infinite past and has reached -1 at present, we can make a counterexample to (S*2). This argument can be formalized as follows:

DA*

(D*1) It is possible that George has always been counting negative integers from the infinite past and has reached -1 at present.

(D*2) If George has always been counting negative integers from the infinite past and has reached -1 at present, then for each negative integer, n, George has counted n.

(D*3) If George has counted each negative integer, then George has by now counted ℵ_0_-many numbers.

Therefore:

(D*4) It is possible that George has by now counted ℵ_0_-many numbers.

According to this scenario, the collection of George’s counting events is an actual infinite whose formation process is already completed by successive addition. This is because the set of all negative integers is an actual infinite, and every negative integer has already been counted by George. As a result, the truth of (D*4) implies that there can exist an actual infinite whose formation process is completed by successive addition. But this contradicts (S**2). So, if DA* is sound, then SAA** would be refuted, and the finitude of the past cannot be established by this argument. However, this reply seems to be wanting for at least two reasons.

First, DA* seems to be less intuitively plausible than DA. The example of counting from the infinite past lacks the intuitive strength that the initial example of counting to the infinite future enjoys. We can easily imagine that someone starts counting at some moment of time and never stops counting. But we cannot easily understand what it means to be always counting without ever starting to count. Our intuitions about the possibility of beginningless counting are not as robust as our intuitions regarding the possibility of an endless counting process. We can easily affirm the latter possibility. But our intuitions are (at least to some extent) resistant to the endorsement of the former possibility. So, it seems that, in this specific regard, the intuitive force of DA* against SAA** is much less than the force of DA against SAA.

Second, and perhaps more importantly, the employment of DA* against SAA** is illegitimate for exactly the same reason that the employment of DA against SAA is illegitimate. DA* cannot be sound unless it is already accepted that the past can have no beginning. This is so because, to maintain the similarity of the counting events of DA* to the events that SAA** is about, the counting events of DA* must happen at a constant rate (i.e., one counting event per a temporal period of a non-zero length). But this means that the truth of (D*1) cannot be guaranteed unless the possible infinitude of the past is already accepted. We cannot affirm that it is possible that George has always been counting negative integers from the infinite past and has reached -1 at present unless we have already accepted that the past is possibly infinite (or, equivalently, that the temporal series of past events can be an actual infinite). This indicates that the possible infinitude of the temporal series of past events is a hidden premise of DA*. Consequently, DA* cannot be used as an objection against SAA**, which is an argument against the possible infinitude of the temporal series of past events. When the question of whether or not the past can be infinite is not yet settled, we cannot criticize an argument for a specific position (regarding that question) by appealing to an argument that simply presupposes the negation of that position. As mentioned before, such an attack is indeed a nonstarter. Thus, it seems that SAA** is not vulnerable to either DA or DA* (albeit for different reasons).

Before closing the paper, it must be clarified that I do not claim my discussion justifies (S**2). The defenders of KCA could propose various arguments in defence of these premises. For example, following a line of argument developed by Andrew Loke, one might say that if the formation process of an actually infinite collection can be completed by successive addition, then the building process of Hilbert’s Hotel can in principle be completed.^16^ However, the existence of Hilbert’s Hotel is impossible (due to the absurdities that it raises).^17^ Therefore, the formation process of an actually infinite collection can never be completed by successive addition. Thus, (S**2) is true, or so a defender of KCA might argue and conclude. Nevertheless, I do not contend that this argument is sound. But even if it is not sound, it is not because it is in tension with DA. Generally, my claim is not that (S**2) is true. Rather, it is that even if (S**2) is false, its falsity cannot be established by appealing to DA.

My discussion clarifies that there is an argument for the finitude of the past from the impossibility of an actually infinite number of successive additions (i.e., SAA**) whose soundness cannot be questioned by either DA or DA*. DA is not effective against SAA** because the collection of George’s future counting events will never be completed by successive addition. So, DA does not provide a counterexample to (S**2). The conclusion of DA is perfectly compatible with the truth of (S**2) and the soundness of SAA** in general. On the other hand, employing DA* against SAA** is illegitimate because DA* simply presupposes the negation of the conclusion of SAA** from the outset. So, if we attack SAA* by DA* we have begged the question of the unsoundness of SAA**. The moral is that neither DA nor DA* can play a decisive role in refuting arguments for past-finitism from the impossibility of an infinite number of successive additions.

## Footnotes

1 See, for example, Craig (1979, 63; 2008, 111).

2 See, for example, Craig (1979, 103) and Craig and Sinclair (2009, 117). Craig seems to presuppose that there is a unique temporal series of events. That is probably why he uses the definite article for ‘temporal series’. To preserve the homogeneity of my presentation with that of Craig, I stick to the same strategy and assume that ‘the temporal series of events’ refers to the series of all holistic events that happen in consecutive temporal intervals of a certain length (e.g., seconds, days, years, etc.). Each holistic event of a specific interval of time can be taken as a composite event which is constituted of all changes that happen in the universe during that period.

3 Dretske (1965).

4 The argument is quoted from Malpass (forthcoming, 3).

5 Craig (2008, 116).

6 As Malpass (forthcoming, 16) says: “Then, because Dretske’s argument establishes the possibility of George counting each natural number, it would follow that such an infinite sequence can be ‘formed by successive addition’.”.

7 See footnote 2.

8 See for example Dretske (1965, 99) and Malpass (forthcoming, 2 and 10).

9 Malpass (forthcoming, Sec. 8).

10 Malpass (forthcoming, 16).

11 Malpass (forthcoming, 16).

12 Malpass (forthcoming, 17).

13 Put differently, if the universe has no beginning, then the temporal series of past events satisfies Craig’s definition of actual infinites that I mentioned earlier in the paper.

14 See, for example, Dretske (1965, Sec. 3) and Malpass (forthcoming, Sec. 6). In a more general context, the subtle difference between *what will be* and what *will have been* is discussed by Malpass and Morriston (2020) in detail.

15 Malpass (forthcoming, Sec. 7).

16 See Loke (2014).

17 Note that Hilbert’s Hotel can be considered a collection which satisfies *Accumulation* because all its rooms are supposed to exist together, regardless of whether or not we endorse presentism. So, a defender of KCA can claim that the crucial difference between the actual infinity of the temporal series of past events and the actual infinity of George’s counting event is that the former entails the possibility of the existence of a collection (e.g., Hilbert’s Hotel) which satisfies *Accumulation* but the latter does not have such absurd consequences.
